# Direct Extraction of Fumaric Acid from *Rhizopus oryzae* Suspensions—Interfacial Mass Transfer

**DOI:** 10.3390/biom11111563

**Published:** 2021-10-21

**Authors:** Dan Cascaval, Anca-Irina Galaction, Alexandra Tucaliuc, Lenuta Kloetzer

**Affiliations:** 1 Cristofor Simionescu Faculty of Chemical Engineering and Environmental Protection, Gheorghe Asachi Technical University, D. Mangeron 73, 700050 Iasi, Romania; dan.cascaval@academic.tuiasi.ro (D.C.); alexandra.tucaliuc@academic.tuiasi.ro (A.T.); 2 Faculty of Medical Bioengineering, Grigore T. Popa University of Medicine and Pharmacy, M. Kogalniceanu 9-13, 700454 Iasi, Romania; anca.galaction@umfiasi.ro

**Keywords:** fumaric acid, Amberlite LA-2, 1-octanol, reactive extraction, mass flow, *Rhizopus oryzae*

## Abstract

Experimental studies on the reactive extraction of fumaric acid with Amberlite LA-2 from *Rhizopus oryzae* suspensions using three solvents with different dielectric constants varying from 9.08 to 1.90 (dichloromethane, n-butyl acetate, and n-heptane, respectively) underlined the particular behavior of the extraction system in the presence of fungal biomass. The interfacial mass flow of the reaction product was found to be significantly affected by the biomass, due mainly to its adsorption onto the phase separation interface, this leading to the appearance of a physical barrier against the solute’s transfer. However, the magnitude of the adsorption phenomenon was found to depend on *Rhizopus oryzae*’s affinity for the solvent phase, which increased significantly from dichloromethane to n-heptane. The negative influence of the biomass on the interfacial mass transfer can be partially counteracted by adding 1-octanol into the organic phase, improving the solvent’s ability to solve the fumaric acid–Amberlite LA-2 complex and simplifying the reactive extraction mechanism, effects that were found to be more important for low-polar solvents. Consequently, for the same mixing intensity, the maximum amplification factor was reached for n-heptane, its value being almost 5–6 times higher than that obtained for dichloromethane and over 2 times higher than that obtained with n-butyl acetate.

## 1. Introduction

Fumaric acid, a naturally occurring organic acid, is an important platform molecule with a broad range of applications in the food industry (a preservative for food and beverages, a base for artificial flavors, a coagulant, a regulator of acidity, an additive for ruminant feed, etc.), healthcare and biomedical fields, and the chemical industry (raw material for the production of organic acids, dyes, mordants, polyalcohols, biodegradable polymers, polyester, and alkyd resins and compounds used in medicine) [1,2]. It is an intermediate in the citric acid cycle and it is widely found in nature, being isolated for the first time from the plant *Fumaria officinalis*. Annually, over 300,000 tons of fumaric acid is consumed, this amount being produced mainly by chemical synthesis starting from benzene, n-butane, or maleic anhydride, but also by fermentation using *Rhizopus* spp., mainly *Rhizopus oryzae* and *Rhizopus arrhizus* [2,3,4,5,6].

The interest in producing fumaric acid by biosynthesis is justified by the large amount of material consumed and the energy required by the chemical technologies. Thus, the cost, yield, and quality of the final product depend on the fluctuating cost of the raw materials and the characteristics of the catalysts used for the production of fumaric acid by a chemical route [5]. Moreover, another reason is the demand for extending the new eco-friendly technologies, namely the low-cost fermentation technologies that use a fungus such as *Rhizopus oryzae* (family Mucoraceae) [6]. The fungal morphology of *Rhizopus oryzae* is one of the main factors influencing the efficiency of fumaric acid’s biosynthesis process. The formation of different fungal morphologies, such as clumps, filamentous mycelia, and pellets, during fermentation is affected by the growth conditions. Thus, the main carbon source is glucose, this being preferred because it is readily metabolized by fungi, while the traditional nitrogen sources are ammonium salts and yeast extract. Small pellets are the preferred morphology for fumaric acid’s fermentation in order to reach the optimal oxygen and mass transfer, these parameters being enhanced due to the low–medium viscosity [2,3,4].

Besides the technological difficulties with the production methods, the downstream processes also raise major problems concerning environmental protection. Currently, the separation of fumaric acid at a large scale is based on ion exchange absorption, precipitation as a calcium salt, electrodialysis, and crystallization [5]. These methods require large amounts of acids and produce severe pollutants, such as acidic wastewater and calcium sulfate sludge [5].

These methods are also applied for the separation of fumaric acids from fermentation broths, with the same problems as those mentioned above. At the same time, fumaric acid induces an important inhibitory phenomenon, its accumulation in the broth leading to the fungal cells’ lysis and, implicitly, to the diminution of the *Rhizopus oryzae* population’s activity. This phenomenon can be avoided by direct removal of the acid during the fermentation process using a method that does not interfere with the fermentation process and exhibits no stress on the cultivated microorganisms. An example of such a separation method is liquid–liquid extraction, which has been successfully tested for the direct separation of some biosynthetic compounds [7]. However, due to the fumaric acid’s dissociation at the fermentation process’s pH value and to the low solubility of fumarate ions in the hydrophobic organic solvents, physical extraction is inefficient [5]. The efficiency of physical extraction can be significantly improved by using extractants that are able to transform the fumaric acid by a chemical reaction into a compound soluble in organic phase. This method is called reactive extraction and it has previously been studied for the purpose of efficiently separating fumaric acid using Amberlite LA-2 dissolved in various solvents with different dielectric constants [8]. The reactive extraction of fumaric acid occurs by an interfacial reaction with salt or the formation of an aminic adduct, depending on the organic phase’s polarity. The reactive extraction yield reached over 95% for solvents with a higher polarity when ammonium fumarate was produced, the process’s efficiency being improved by adding a phase modifier [8].

On the basis of the information resulting from studies on the reactive extraction of fumaric acid from pure aqueous solutions and simulated broths without biomass [8], an investigation of this acid’s separation by the same technique from *Rhizopus oryzae* suspensions was performed. Therefore, the influence of the solvent dielectric constant and 1-octanol addition on the interfacial mass transfer rate of fumaric acid was analyzed in direct relation to the fungal biomass’s concentration. Because the presence of biomass could affect the mass transfer between the aqueous and organic phases and, implicitly, the reactive extraction efficiency, the results are discussed in comparison to those obtained for simulated broths, without biomass, but possessing similar apparent viscosities.

## 2. Materials and Methods

The experimental program included two distinct stages. Initially, the limiting steps of the reactive extraction process of fumaric acid were analyzed. In the second step, the interfacial mass flows of fumaric acid were determined in relation to the above-mentioned factors.

**Figure 1:**
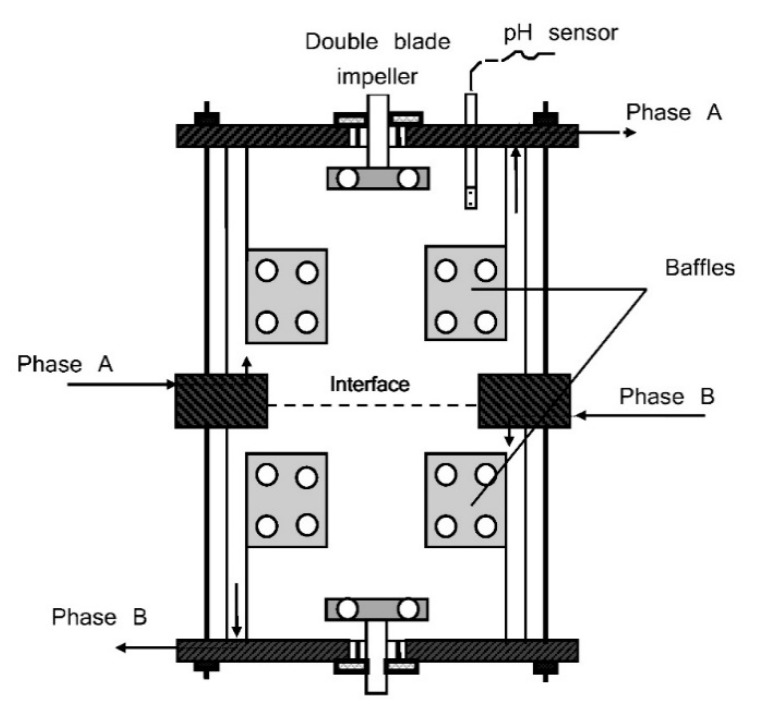
Extraction cell.

The experimental equipment consisted of an extraction cell of modified Lewis type previously described in [9] (Figure 1). The extraction cell consisted of two compartments of 750 mL each and was made of stainless steel and glass. The experiments were carried out in a continuous operating regime, the two phases being separately fed with an identical volumetric flow of 2.5 L/h. The aqueous and organic phases were independently stirred using perforated blade impellers. The impellers’ rotation speed varied from 0 to 1200 rpm. The interface that separated the aqueous and organic phases was kept flat, its area measuring 2.83 × 10^−3^ m^2^. The extraction temperature was maintained at 25 °C during the experiments.

The initial concentration of fumaric acid in the aqueous phase was 5 g/L (0.044 M). The aqueous phase consisted of suspensions of *Rhizopus oryzae* having a fungal biomass concentration of between 4 and 24 g/L d.w., this range corresponding to the biomass accumulation that occurs during a real fermentation process [10]. The considered values of biomass concentration are also related to the viscosity of simulated broths previously studied in [10]. Because of the difficulty of measuring in situ the viscosity of the fungal suspensions during the experiments, the viscosity was measured before and after each experiment by means of a HAAKE Viscotester 6 Plus instrument (PSL Systemtechnik GmbH, Osterode am Harz, Germany). The viscosity was measured at the temperature of 25 °C, any viscosity changes or mechanical lysis of biomass being recorded during the extraction experiments.

The reactive extraction was carried out using Amberlite LA-2 (molecular weight, 360.3 g/mol; density, 0.83 g/cm^3^; temperature, 25 °C) (Serva, Heidelberg, Germany, p.a. ≥ 95%) dissolved in three solvents with different dielectric constants, namely dichloromethane (ε = 9.08) (Sigma-Aldrich Laborchemikalien GmbH, Seelze, Germany, p.a. ≥ 99.9%), n-butyl acetate (ε = 5.01) (Sigma-Aldrich Chemie GmbH, Steinheim, Germany, p.a. ≥ 99%), and n-heptane (ε = 1.90) (Carl Roth GmbH &Co. KG, Karlsruhe, Germany, p.a. ≥ 99%) [11]. The organic phase was the solvent or a mixture of the solvent and 1-octanol (Sigma-Aldrich, Inc., St. Louis, MO, USA, p.a. ≥ 99%) added as a phase modifier (dielectric constant ε = 10.3 at 25 °C [11]). The volumetric fraction of 1-octanol in the organic phase was 0.10. The Amberlite LA-2’s concentration in the organic phase was 60 g/L (0.16 M). Depending on the relative densities of the two phases according to Figure 1, the organic phase could be Phase A, while the *Rhizopus oryzae* suspension could be Phase B, or vice versa.

The pH value of the aqueous phase was maintained at 2, this being the pH value corresponding to the maximum extraction yield [8]. The pH’s adjustment to the prescribed value was made with a solution of 3% sulfuric acid (Sigma-Aldrich Chemie GmbH, Steinheim, Germany, p.a. ≥ 99.9%). The pH value was recorded throughout each extraction experiment, and any pH change was recorded.

The extraction process was analyzed by means of the fumaric acid interfacial mass flow, n, from the aqueous phase to the solvent. To calculate this parameter, the fumaric acid concentrations in the initial aqueous phase and in the raffinate were measured. To determine the acid concentration in the organic phase, the mass balance was used. The fumaric acid concentration was determined by the HPLC technique (UltiMate 3000 Dionex, Dionex Softron GmbH, Germering, Germany) with an Acclaim^TM^ OA column (diameter, 4 mm; length, 150 mm; porous particles, 5 µm, Thermo Fisher Scientific, Sunnyvale, CA, USA) and a UV detector at 210 nm [7]. The mobile phase was a solution of 100 nM sodium sulfate, its pH value being adjusted to 2.65 with methanesulfonic acid (Sigma-Aldrich Chemie GmbH, Steinheim, Germany, p.a. ≥ 99.5%). The flow rate of the mobile phase was 0.6 mL/min. The analysis was carried out at 30 °C. Each experiment was repeated three times using identical conditions, the average value of the discussed parameters being considered. The maximum experimental error was ± 6.28%.

## 3. Results

Experiments previously carried out on the separation of fumaric acid from pure and viscous aqueous solutions by reactive extraction indicate that the process efficiency is controlled by the pH value and viscosity of the aqueous phase, the Amberlite LA-2’s concentration in the organic phase, and the organic phase’s polarity [8]. Among these factors, the viscosity and the polarity exhibit the most important influence, both by enhancing the resistance to the acid diffusion from the aqueous solution to the solvent and by determining the extraction mechanism and the interfacial equilibrium [8].

The interfacial reaction between fumaric acid and Amberlite LA-2 can be described by the general mechanism [8]:

R(COOH)_2(aq)_ + p Q_(o)_ ⇄ R(COOH)_2_·Q_p(o)_

where R(COOH)_2_ denotes the fumaric acid and Q denotes the extractant. The value of the stoichiometric coefficient p depends on the solvent phase dielectric constant. Therefore, p = 2 for n-butyl acetate and n-heptane without 1-octanol, and p = 1 for dichloromethane and extraction systems to which 1-octanol has been added in the three organic phases [8].

As was observed in previous studies on the direct extraction of other carboxylic acids from their specific fermentation broths, the presence of biomass significantly affects the efficiency of reactive extraction due to the following phenomena [7,9]:

- the biomass’s adsorption onto the interface separating the aqueous phase from the solvent induces the appearance of a physical barrier between the two phases, implicitly inducing an additional resistance to the interfacial transfer of the solute;
- the presence of biomass leads to an increase in the apparent viscosity of the aqueous phase, thus enhancing the diffusional resistance to the solute’s transfer;
- during the extraction process, mechanical lysis of the biomass could be produced by the shear stress generated by mixing the two phases. This phenomenon allows for the release into the aqueous phase of cytoplasmic compounds (amino acids, organic acids, or proteins) that could be co-extracted or could precipitate.

Because the aim of this work is to quantify the influence of *Rhizopus oryzae* biomass on the reactive extraction of fumaric acid, the experiments were carried out in a manner identical to that considered in the previous studies on this acid’s reactive extraction from pure aqueous solutions and simulated broths, namely viscous solutions without biomass.

**Figure 2:**
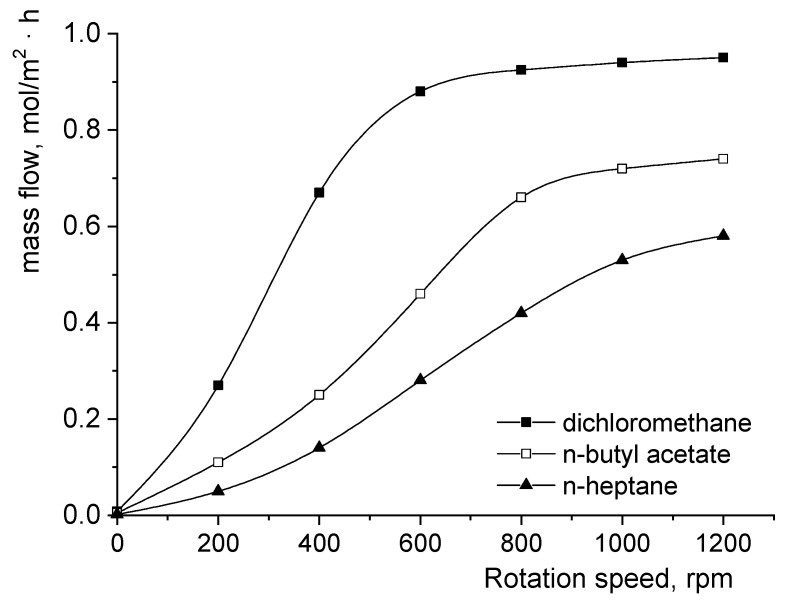
Influence of the impellers’ rotation speed on the fumaric acid mass flow from the pure aqueous phase to the organic phase without 1-octanol.

In order to compare the importance of the diffusional or kinetic resistances of the fumaric acid’s transfer from the aqueous phase to the organic phase, the dependence between the acid interfacial mass flow and the impellers’ rotation speed was plotted and is shown in Figure 2. These dependencies were comparatively analyzed using the reactive extraction of fumaric acid from a pure aqueous solution in solvents without 1-octanol.

The superior dielectric constant of dichloromethane enhances this solvent’s capacity to solve the interfacial complex formed between fumaric acid and Amberlite LA-2. Consequently, for the entire experimental range of rotation speeds, the highest mass flows were reached for dichloromethane. Based on the solubilization power of the solvent phases, the decreasing order of the mass flow values corresponds to the decreasing order of polarity, namely from dichloromethane to n-heptane. However, apart from the variations in mass flow, Figure 2 also has to be analyzed by considering the mechanism of the interfacial reaction between the solute and the extractant. Therefore, in the case of dichloromethane without 1-octanol, the participation of one molecule of each reactant in the interfacial reaction together with the highest solvent polarity justified the lowest critical rotation speed (defined as the rotation speed value that corresponds to the transition from the diffusional regime to the kinetic regime [8]) (600 rpm, Figure 2).

The dielectric constants of n-butyl acetate and n-heptane are significantly lower than that of dichloromethane. In these circumstances, the importance of diffusional resistance is amplified compared with that of kinetic resistance. For these reasons, the rotation speed ranges related to the diffusional regime extend from 0 to over 800 rpm for n-butyl acetate and exceed the considered maximum rotation speed for n-heptane (Figure 2). Obviously, the critical rotation speed for n-butyl acetate is lower than that for n-heptane due to its superior polarity and, implicitly, to the lower resistance to interfacial diffusion of the formed complex compared with n-heptane.

**Figure 3:**
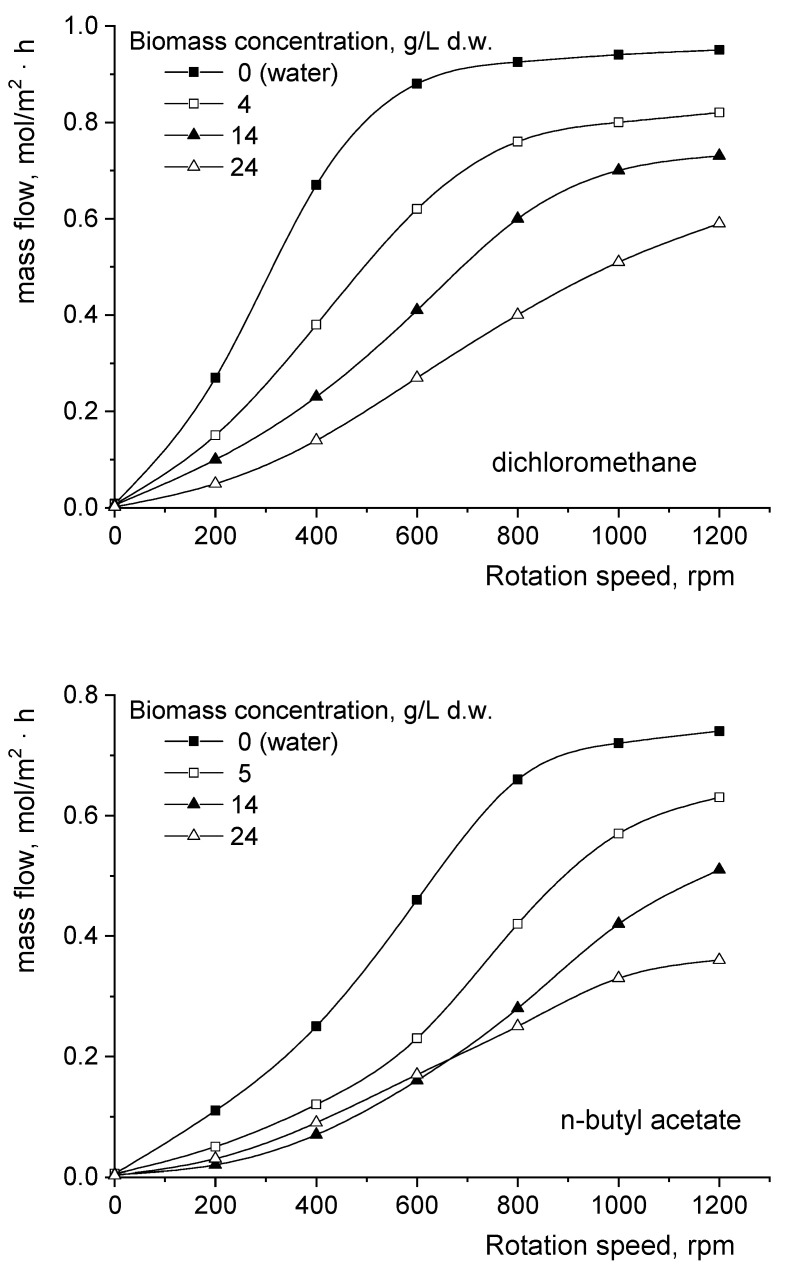

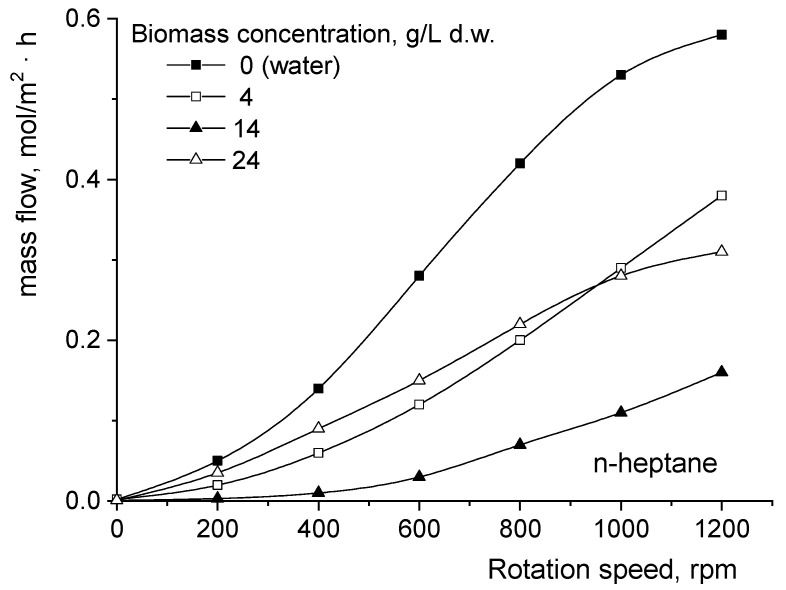
Influence of the impellers’ rotation speed on the fumaric acid mass flow from *Rhizopus oryzae* suspensions to the organic phase without 1-octanol.

Figure 3 indicates that the extracted compound mass flows are reduced in the presence of *Rhizopus oryzae* biomass for all studied solvents. At the same time, the presence and concentration of fungal biomass modify the critical rotation speed value. However, the reactive extraction system’s behavior has to be correlated to the nature of the solvent phase, namely to the affinity of *Rhizopus oryzae* for this phase.

Therefore, for dichloromethane, the fungal biomass accumulation leads to an increase in the critical rotation speed from 600 rpm for water (0 g/L d.w. *Rhizopus oryzae*) to a value that exceeds the limit considered in the experiments (1200 rpm) for 24 g/L d.w. *Rhizopus oryzae*. This variation suggests the amplification of the resistance to the diffusion of the compound formed by the reaction between fumaric acid and the extractant from the aqueous phase to the organic phase due to the accumulation of fungal biomass. Because no affinity of the fungus for dichloromethane has been previously reported in the literature, in this case the observed effect is the result of the increase in the aqueous phase’s viscosity.

For the other two solvents, the amount of *Rhizopus oryzae* does not represent the singular parameter influencing the extent of the rotation speed range corresponding to the extraction’s development in the diffusional or kinetic regime. In these cases, another important factor is the fungus’s affinity for the solvent phase and the variation in this affinity during the biomass’s accumulation.

Fungi possess different affinities for esters compared with hydrocarbons, this leading to different variations in the mass flow with the rotation speed for n-butyl acetate compared with n-heptane for similar biomass concentrations (Figure 3). Although for both solvents the biomass accumulation determines the increase in the critical rotation speed value, for the above-discussed reason, the order of the mass flow decrease by increasing the *Rhizopus oryzae* amount for a given rotation speed depends on the nature of the organic phase.

As the affinity of fungi for esters is rather weak, these compounds could be used as a substrate, but their consumption is not efficient [12,13]. However, the affinity for n-butyl acetate induces the adsorption of *Rhizopus oryzae* onto the separation interface between the aqueous and solvent phases. The fungus’s adsorption onto the interface leads to the appearance of a physical barrier between the two phases and, consequently, a reduction in the solute mass flow. The intensification of mixing promotes the biomass’s desorption and dispersion into the aqueous phase, this counteracting the interface blockage. The magnitude of the blockage phenomenon is more important during the fungal growth period, when its amount is reduced. For this reason, from Figure 3 it can be observed that at rotation speeds below 700 rpm, the values of the mass flow recorded for 24 g/L d.w. *Rhizopus oryzae* are higher than those for 14 g/L d.w. *Rhizopus oryzae*. Moreover, the shapes of the variations plotted for 4 and 24 g/L d.w. *Rhizopus oryzae* are rather similar, indicating the attenuation of the rotation speed’s influence over 1000 rpm. This effect cannot be observed for 14 g/L d.w. *Rhizopus oryzae* as a result of the greater interfacial adsorption of biomass. However, over 700 rpm, due to the above-discussed positive effect of the intensification of mixing, the decreasing order of mass flow respects the increase in the fungal biomass concentration.

Instead, *Rhizopus oryzae* exhibits a stronger affinity for hydrocarbons, as the interfacial adsorption of fungal biomass is more pronounced in the case of n-heptane [12]. Similarly to esters, the affinity of fungi for hydrocarbons is more important at the beginning of their growth, at a low biomass concentration, the accumulation of fungal biomass inducing their desorption from the interface. Compared with n-butyl acetate, due to the stronger affinity of *Rhizopus oryzae* for n-heptane, the positive influence of the intensification of mixing on the interfacial desorption and, implicitly, on the interfacial mass transfer of the solute is diminished. As a consequence of the above-described aspects, Figure 3 indicates that for rotation speeds below 1000 rpm, the highest rates of interfacial mass transfer of the complex are reached for the most-concentrated *Rhizopus oryzae* suspensions. Thus, for superior rotation speed values the highest rates of interfacial mass transfer corresponding to 24 g/L d.w. *Rhizopus oryzae* decrease to below those obtained for 4 g/L d.w. *Rhizopus oryzae* but remain superior to those for 14 g/L d.w. *Rhizopus oryzae*.

These mechanisms through which the fungal biomass influences the interfacial transfer of the solute from the aqueous phase to the solvent phase were confirmed by analyzing the variation in the reduction factor R by intensifying the mixing. The factor R was previously defined as the ratio between the solute mass flows obtained for reactive extraction from real broths and those obtained for reactive extraction from simulated broths with similar viscosities [8].

**Figure 4:**
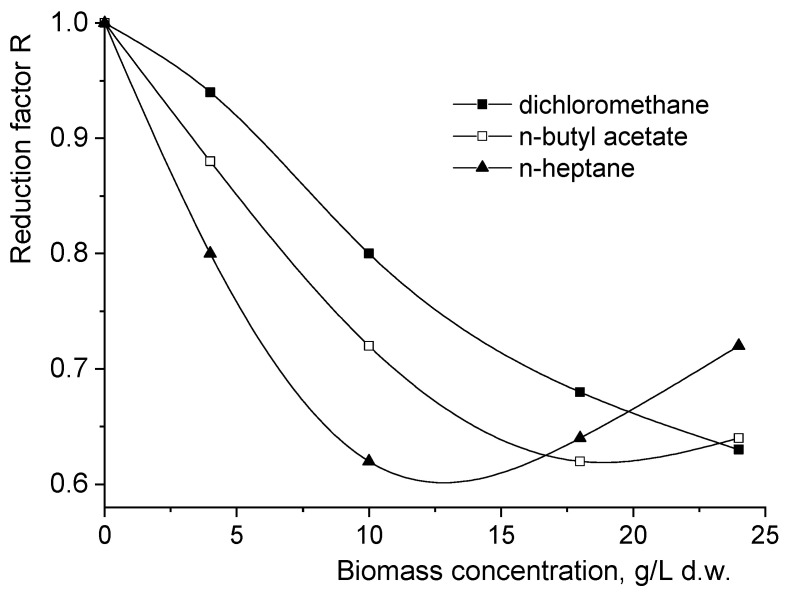
Influence of *Rhizopus oryzae* concentration on the reduction factor for the organic phase without 1-octanol (rotation speed = 1000 rpm).

Without exception, Figure 4 suggests that the presence of fungal biomass exhibits an important negative influence on interfacial mass flows. For this reason, the values of the reduction factor R are below 1 for the entire considered range of *Rhizopus oryzae* concentrations, the minimum being 0.62–0.63 for all studied solvents. However, the shapes of the plotted variations and the biomass concentration corresponding to the minimum factor R value depend on the solvent type, particularly on the fungus’s affinity for the organic phase.

According to Figure 4, the accumulation of *Rhizopus oryzae* determines the continuous reduction in R only for dichloromethane, the minimum value of R = 0.62 being reached for the most-concentrated fungal suspensions considered in the experiments. Due to the fungus’s affinity for n-butyl acetate and n-heptane and, implicitly, to the additional hindrance of the solute’s interfacial transfer by biomass adsorption, the reduction in factor R is initially more pronounced for these two solvents compared with dichloromethane. On the basis of the above-discussed reasons, at higher biomass concentrations, the interface blockage is diminished by desorption, this leading to an increase in the factor R. The different affinities, amplitudes of mixing intensification effects on interfacial mass transfer, and, consequently, magnitudes of desorption phenomenon effects on interfacial mass transfer induce different increases in R for n-butyl acetate compared with n-heptane. Therefore, the factor R for n-butyl acetate reaches its minimum value of 0.62 for 18 g/L d.w. *Rhizopus oryzae*, increasing then slowly to 0.64. For n-heptane, the above-mentioned phenomena being more pronounced, the minimum value of factor R corresponds to 10 g/L d.w. *Rhizopus oryzae* and increases finally to a value that exceeds those recorded for the other two solvents (0.72).

As was previously reported, the yield of reactive extraction is enhanced by the addition of a more polar organic liquid in the solvent phase [8,9]. This effect is more pronounced for lower-polarity solvents, being the result of the improvement in the organic phase’s capacity to solve the polar molecules. Because the added compound exhibits a favorable influence both on the organic phase’s polarity and on the breakage of the stable “third-phase” emulsion specific to extraction systems that use amines as extractants, it has been called a “polarity or phase modifier” [8]. Generally, the phase modifier is a long-chain alcohol containing at least eight carbon atoms in the aliphatic chain, 1-octanol being considered for this purpose in these experiments.

**Figure 5:**
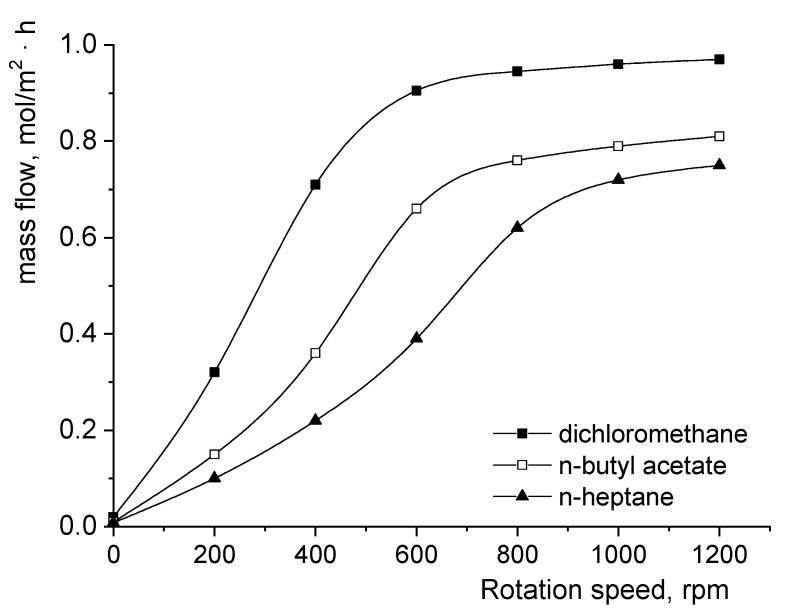
Influence of the impellers’ rotation speed on the fumaric acid mass flow from the pure aqueous phase to the organic phase with 1-octanol.

According to Figure 5, the addition of 1-octanol intensifies the interfacial mass transfer, this reducing the value of the critical rotation speed. However, the amplitude of this effect varies significantly from one solvent to another depending on the dielectric constant. Therefore, due to it having the highest polarity among the studied solvents, the least important influence of alcohol on the mass flow was reached for dichloromethane (the critical rotation speed value remains 600 rpm, and the mass flow related to 1000 rpm increases negligibly by 2%). The influence of 1-octanol becomes more pronounced at lower mixing intensities because the presence of the alcohol partially counteracts the lower diffusional rate promoted by the lower degree of turbulence (at 200 rpm, the solute mass flow increases by almost 20%).

The positive effect of 1-octanol is more obvious for n-butyl acetate and n-heptane, its magnitude being stronger for n-heptane. For n-butyl acetate, the critical rotation speed value is reduced to 600 rpm, similar to that for dichloromethane, while for n-heptane the critical rotation speed value is reduced to 800 rpm. At 1000 rpm, the interfacial mass flow of the extracted complex is increased by 10% for n-butyl acetate and by 17% for n-heptane, this difference being amplified at 200 rpm (the solute mass flow increases by 36% for n-butyl acetate and by 200% for n-heptane) (Figure 5).

The above-discussed variations suggest the relative enhancement of the kinetic resistance compared with that induced by the diffusional process in the overall reactive extraction process of fumaric acid. On the other hand, for n-butyl acetate and n-heptane the mechanism of reactive extraction is simplified in the presence of 1-octanol, the chemical structure of the extracted compound becoming R(COOH)_2_.Q [8]. Because only one molecule of fumaric acid and, respectively, Amberlite LA-2 participate in the formation of the extracted compound, the rate of the interfacial chemical reaction is increased and, consequently, the kinetic resistance is diminished. Consequently, by dissolving 1-octanol into the solvent phase, the diffusional and kinetic resistances are diminished and, implicitly, the solute interfacial mass flow is intensified. Due to its having the lowest polarity, this effect is considerably amplified for n-heptane.

**Figure 6:**
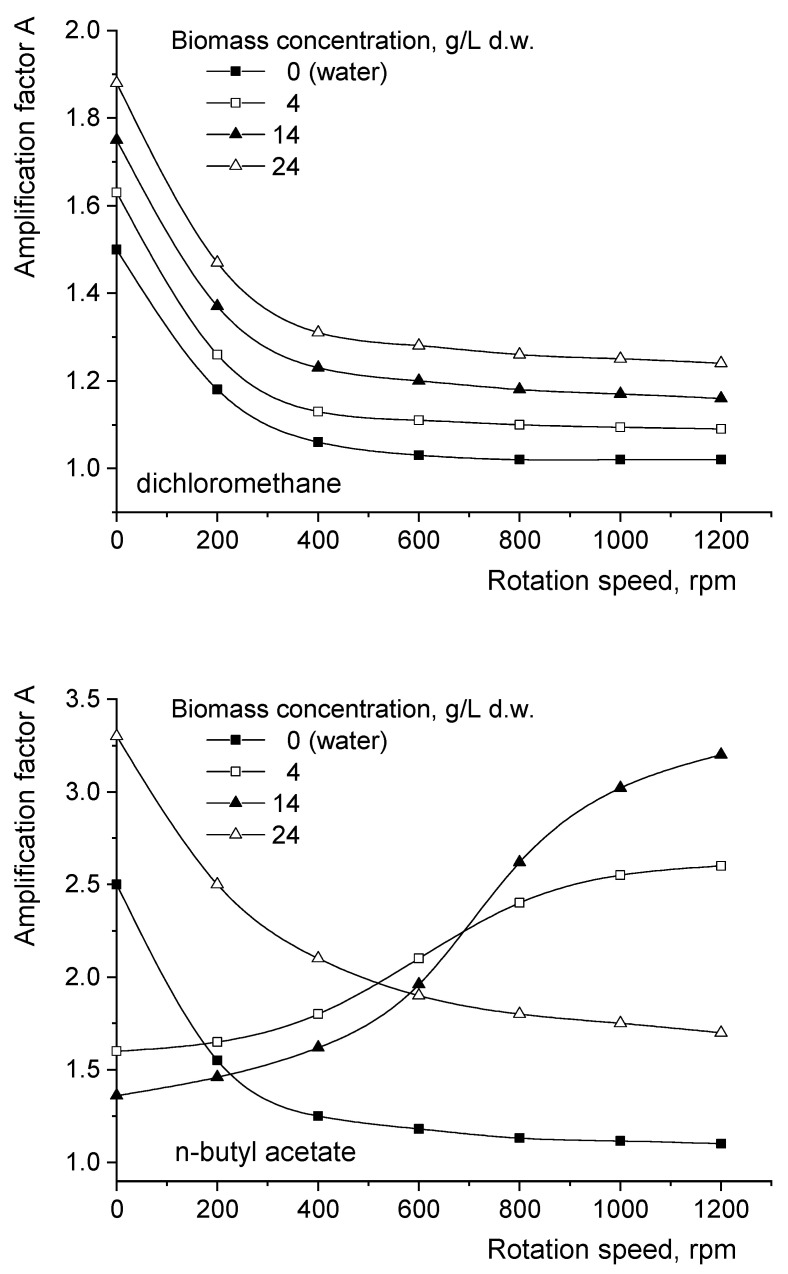

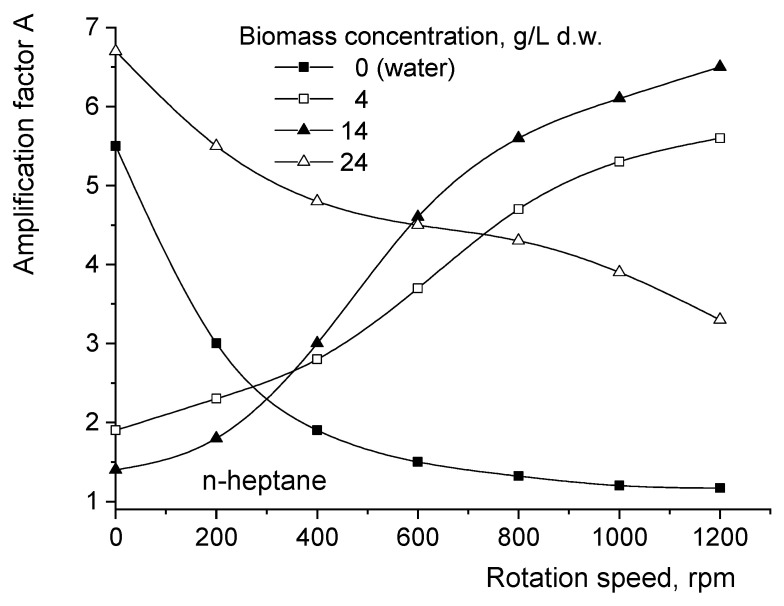
Influence of the impellers’ rotation speed on the amplification factor.

The positive influence of 1-octanol on the reactive extraction efficiency can be described by means of the amplification factor A, previously defined as the ratio between the interfacial mass flows with and without 1-octanol, respectively [9]. According to the variations plotted in Figure 6, in all cases the factor A is over 1, confirming that the addition of 1-octanol exhibits a positive effect on the extraction efficiency, an effect that becomes more important as the solvent dielectric constant is reduced. Furthermore, this influence of alcohol has to be analyzed in relation to the presence of fungal biomass, particularly in relation to its affinity for the solvent phase.

For the reactive extraction of fumaric acid from pure aqueous solutions, Figure 6 indicates the continuous reduction in factor A with the intensification of the mixing for all the used solvents. This variation can be divided into two domains: for lower rotation speeds, the decrease in factor A is significant, becoming much less pronounced over a certain value of the impellers’ rotation speed. This variation is the result of the modification of the relative contribution of the alcohol vs. the turbulence induced by mixing to the diffusion and solubilization of the solute. At lower rotation speeds, the improvement in the organic phase’s capacity to solubilize the solute by the addition of 1-octanol represents the main factor that controls the interfacial mass transfer. By increasing the rotation speed, the role of the mixing intensity in promoting high diffusional and solubilization rates is amplified, this leading to mass flow values close to those recorded for the reactive extraction in the absence and, respectively, the presence of 1-octanol. The rotation speed value corresponding to the change in the factor A variation slope decreases more slowly or remains at a constant value with the intensification of mixing. Its variation depends on the solvent type and can be correlated to the critical rotation speed value. In fact, over the range of critical rotation speed values (600 rpm for dichloromethane, 800 rpm for n-butyl acetate, and 1000 rpm for n-heptane), the kinetic regime replaces the diffusional regime, the role of diffusion as a limiting step being canceled. As was mentioned above, the value of A can be increased by reducing the solvent’s polarity (at 1000 rpm, factor A is 1.02 for dichloromethane, 1.10 for n-butyl acetate, and 1.17 for n-heptane) (Figure 6).

Depending on the fungal biomass’s affinity for the organic phase, particularly on its ability to be adsorbed onto the interface between the two extraction phases, the presence and accumulation of *Rhizopus oryzae* biomass could significantly modify the shapes of the dependencies between factor A and the rotation speed. Moreover, the values of the amplification factor for fumaric acid’s extraction from *Rhizopus oryzae* suspensions generally exceed those obtained from its extraction from pure solutions (Figure 6).

For dichloromethane, due to the negligible affinity of the fungus for solvent, the variations in factor A for different concentrations of fungal biomass are similar to that recorded for pure aqueous solution. The presence of 1-octanol exhibits a more pronounced effect at higher *Rhizopus oryzae* amounts; at 1000 rpm, factor A increases from 1.02 for pure solution to 1.25 for suspensions with 24 g/L d.w. biomass.

In the case of n-butyl acetate, only at higher fungal biomass concentrations is the variation in the amplification factor with the rotation speed similar to that plotted for the pure solution of fumaric acid, but the values are significantly higher due to the more pronounced influence of 1-octanol addition to solvents with a lower polarity (at 1000 rpm, factor A increased from 1.10 for the pure solution to 1.75 for suspensions with 24 g/L d.w. biomass) (Figure 6). As was previously discussed, this behavior is a consequence of the lack of biomass adsorption onto the interface in the advanced stages of biomass growth.

The shapes of these variations are completely different for lower biomass concentrations. As can be seen from Figure 6, for 4 g/L d.w. *Rhizopus oryzae* biomass, the factor A is increased by the intensification of mixing, this variation being less important for rotation speeds below 400 rpm and over 800 rpm. The role of mixing in the increase in the amplification factor is more pronounced in the case of biomass adsorption onto the interface due to its positive effect on promoting desorption and, implicitly, on the release of the interfacial area available for solute mass transfer. This effect is cumulative with that induced by the addition of alcohol on the solute solubilization. Obviously, the positive influence of mixing intensity on biomass desorption is less important at lower rotation speeds, below 400 rpm, and in the kinetic regime, over 800 rpm.

The accumulation of *Rhizopus oryzae* does not modify the general shape of the above-discussed variation. However, for 14 g/L d.w. *Rhizopus oryzae*, due to the higher amount of fungal biomass that adsorbed onto the interface, the effects induced by 1-octanol and mixing are attenuated for rotation speeds below 700 rpm, the value of factor A being inferior to that corresponding to 4 g/L d.w. *Rhizopus oryzae* (Figure 6). Above this rotation speed, the magnitude of the cumulative effects of the presence of alcohol and the mixing intensity becomes more important for 14 g/L d.w. *Rhizopus oryzae* and the value of factor A increases strongly. As a result of the more pronounced influence of mixing intensity on the efficiency of fumaric acid’s extraction from fungal suspensions exhibiting affinity for the separation interface, at higher rotation speeds the highest values of the amplification factor are reached for *Rhizopus oryzae* with intermediate concentrations (at 1000 rpm, the value of factor A is 2.55 for 4 g/L d.w. *Rhizopus oryzae* and 3.02 for 14 g/L d.w. *Rhizopus oryzae*).

The reasons for the behavior of the extraction system containing n-butyl acetate remain valid also for extraction with n-heptane. However, as a result of both the lowest dielectric constant of n-heptane and the stronger affinity of *Rhizopus oryzae* for this phase, the above-discussed phenomena become more pronounced and the differences between the system with the pure solution and those containing fungal biomass are amplified. As a consequence of the increased magnitude of the cumulative influences, the values of factor A obtained for 14 g/L d.w. *Rhizopus oryzae* exceed those for more concentrated fungal suspensions for rotation speeds superior to 400 rpm, but not to 700 rpm as in the case of extraction with n-butyl acetate. Moreover, the values of factor A are considerably higher than those obtained for dichloromethane and n-butyl acetate. For a rotation speed of 1000 rpm, the maximum amplification factor for n-heptane corresponds to 14 g/L d.w. *Rhizopus oryzae* and is 5.55 times higher than the maximum value recorded for dichloromethane and about 2.15 times higher than that recorded for n-butyl acetate at the same biomass concentration (Figure 6).

To mathematically describe the cumulative influences of solvent polarity, mixing intensity, and *Rhizopus oryzae* concentration on the solute interfacial mass flow from the fungal suspension to the organic phase, we propose two models for both extraction systems, namely without and with 1-octanol. To respect the diffusional regime for all studied solvents, in the absence or the presence of alcohol, these mathematical expressions are valid for rotation speeds up to 800 rpm: without 1-octanol

(1) $$n=e^{0.164\cdot \varepsilon }\cdot \frac{N^{0.273\cdot \varepsilon }}{C_{X}{}^{1.665}}$$

with 1-octanol

(2) $$n=e^{2.23\cdot 10^{-2}\cdot \varepsilon }\cdot \frac{N^{3.54\cdot 10^{-2}\cdot \varepsilon }}{C_{X}{}^{0.743}}$$

## 4. Conclusions

This study of interfacial mass transfer in direct extraction of fumaric acid from *Rhizopus oryzae* suspensions with Amberlite LA-2 in three solvents with different dielectric constants (dichloromethane, n-butyl acetate, and n-heptane) revealed particular behaviors compared with extraction from pure aqueous or viscous solutions without biomass. The experiments were comparatively carried out for a solvent phase containing or not containing 1-octanol as a phase modifier.

The influence of the biomass is the result of the cumulative phenomena of the increasing viscosity of the aqueous phase and of the appearance of a physical barrier against the solute transfer due to the biomass adsorption onto the interface. The relative magnitude of the two phenomena depends on *Rhizopus oryzae*’s affinity for the solvent phase, which becomes stronger from dichloromethane to n-heptane.

Although in all experimental systems the fungal biomass exhibited an important negative influence on the interfacial mass flow, this effect can be partially counteracted by adding 1-octanol to the organic phase. The 1-octanol improves the solvent’s ability to solve the fumaric acid–Amberlite LA-2 complex formed at the interface, an effect important for solvents with a lower polarity (n-butyl acetate and n-heptane). Moreover, the addition of 1-octanol simplifies the mechanism of the chemical reaction between the acid and the extractant.

The positive effect of 1-octanol addition can be amplified by lowering the solvent’s polarity and increasing the biomass’s affinity for the organic phase. Therefore, at 1000 rpm, the maximum amplification factor was reached for n-heptane (6.5), while the lowest amplification factor was reached for dichloromethane (1.25) for similar fungal biomass concentrations in the aqueous phase.

For the diffusional regime, namely for rotation speeds up to 800 rpm, the discussed influences of solvent polarity, rotation speed, and biomass concentration on the interfacial mass flow of the complex formed by the reaction between fumaric acid and Amberlite LA-2 were described in two mathematical expressions adequate for extraction systems without and with 1-octanol, respectively. These models offer a good degree of concordance with the experimental data, the average deviations being ±6.22% in the absence of alcohol and ±8.46% in the presence of alcohol.

## Data Availability

Not applicable.

## References

[1] Sebastian J., Hegde K., Kumar P., Rouissi T., Brar S.K. (2019). Bioproduction of fumaric acid: An insight into microbial strain improvement strategies. Crit. Rev. Biotechnol..

[2] Ilica R.A., Kloetzer L., Galaction A.I., Cascaval D. (2019). Fumaric acid, production and separation. Biotechnol. Lett..

[3] Goldberg I., Rokem J.S., Pines O.J. (2006). Organic acids: Old metabolites, new themes. Chem. Technol. Biotechnol..

[4] Roa Engel C.A., Straathof A.J.J., Zijlmans T.W., van Gulik W.M., van der Wielen L.A.M. (2008). Fumaric acid production by fermentation. Appl. Microbiol. Biotechnol..

[5] Brar S.K., Sarma S.J., Pakshirajan K. (2016). Platform Chemical Biorefinery: Future Green Chemistry.

[6] Swart R.M., le Roux F., Naude A., de Jongh N.W., Nicol W. (2020). Fumarate production with Rhizopus oryzae: Utilizing the Crabtree effect to minimise ethanol by-product formation. Biotechnol. Biofuels.

[7] Caşcaval D., Cârlescu A., Galaction A.I., Turnea M. (2013). Study on biomass impact on the reactive extraction of succinic acid from Actinobacillus succinogenes suspensions. Ind. Eng. Chem. Res..

[8] Kloetzer L., Ilica R.A., Cascaval D., Galaction A.I. (2019). Separation of fumaric acid by amine extraction without and with 1-octanol as phase modifier. Sep. Purif. Technol..

[9] Galaction A.I., Carlescu A., Turnea M., Caşcaval D. (2012). Direct extraction of propionic acid from Propionibacterium acidipropionici broths with tri-n-octylamine. Chem. Eng. Technol..

[10] Galaction A.I., Tucaliuc A., Ciobanu C., Caşcaval D. (2020). Fumaric acid production by Rhyzopus oryzae in presence of n-dodecane as oxygen-vector. Biochem. Eng. J..

[11] Weast R.C. (1974). Handbook of Chemistry and Physiscs.

[12] Leone R., Breuil C. (1998). Filamentous fungi can degrade aspen steryl esters and waxes. Int. Biodeterior. Biodegrad..

[13] Galaction A.I., Caşcaval D., Oniscu C., Turnea M. (2005). Evaluation and modeling of the aerobic stirred bioreactor performances for fungus broths. Chem. Biochem. Eng. Quart..

